# Multiplexed gene expression analysis of HLA class II-associated podoconiosis implicates chronic immune activation in its pathogenesis

**DOI:** 10.1093/trstmh/traa107

**Published:** 2020-10-24

**Authors:** Diana R Alcantara, Christopher I Jones, Daniel M Altmann, Rosemary J Boyton, Muzlifah Haniffa, Melanie J Newport

**Affiliations:** Brighton and Sussex Centre for Global Health Research, Department of Global Health & Infection, Brighton & Sussex Medical School, University of Sussex, Falmer, Brighton BN1 9PX, UK; Department of Primary Care and Public Health, Brighton & Sussex Medical School, University of Sussex, Falmer, Brighton BN1 9PX, UK; Department of Immunology and Inflammation, Faculty of Medicine, Hammersmith Hospital, Imperial College London, London W12 0NN, UK; Department of Infectious Disease, Faculty of Medicine, Hammersmith Hospital, Imperial College London, London W12 0NN, UK; Institute of Cellular Medicine, Newcastle University, Newcastle upon Tyne, UK; Brighton and Sussex Centre for Global Health Research, Department of Global Health & Infection, Brighton & Sussex Medical School, University of Sussex, Falmer, Brighton BN1 9PX, UK

**Keywords:** archive, gene expression, immunology, lymph nodes, NanoString, podoconiosis

## Abstract

**Background:**

Podoconiosis is a tropical lymphoedema of the leg resulting from barefoot exposure to irritant volcanic soils. Approximately 4 million people are affected, mainly in African highland regions. The pathogenesis of this neglected tropical disease is still largely unknown, although HLA class II (HLAII) polymorphisms are associated with the disease.

**Methods:**

NanoString technology was used to assess expression of 579 immune-related genes in formalin-fixed and paraffin-embedded lymph node archival samples from podoconiosis patients and unaffected controls.

**Results:**

Forty-eight genes were upregulated and 21 downregulated in podoconiosis samples compared with controls. Gene ontology analysis showed differentially expressed genes to be closely related to major histocompatibility complex protein, cytokine and TNF receptor binding genes. Pathway enrichment analysis revealed involvement of lymphocyte activation, adaptive immunity, cytokine signalling, antigen processing and the IL-12 pathways.

**Conclusions:**

This exploratory study reports a multiplex gene expression analysis in podoconiosis and shows upregulation of pro-inflammatory transcripts compatible with the notion of local, chronic immune activation in this HLAII-associated disease. Implicated pathways will inform future research into podoconiosis immunopathogenesis.

## Introduction

Podoconiosis, also known as endemic non-filarial elephantiasis, is a progressive lymphoedema of the lower limb observed in people living and working barefoot on red clay soils of volcanic origin.^1^ Recent evaluation of the worldwide distribution and burden of podoconiosis identified its presence in 32 countries, with tropical African regions being the worst affected.^2,3^ Globally, approximately 4 million people have podoconiosis. In Ethiopia alone, it is estimated that approximately 1.5 million people are affected, making it the country with the highest number of podoconiosis cases. Despite its considerable personal, social and economic burden, podoconiosis remains a relatively unknown and neglected tropical disease.^4^

The pathogenesis of podoconiosis is not yet completely understood. It is a non-infectious, non-communicable condition that develops over a period of several years. Early research on podoconiosis was conducted by Ernest Price in the 1970s and 1980s,^1^ working primarily in Ethiopia. Price was the first to propose the hypothesis that toxic mineral microparticles found in endemic soils cross the plantar skin and are engulfed by macrophages in the lower limb lymphatics. The resulting fibrogenic inflammatory response causes obstruction of lymphatic drainage. This in turn leads to swelling of the feet and legs, which progresses to painful and debilitating lymphoedema, sometimes accompanied by nodular skin changes and hyperkeratosis.^1^ Femoral lymphadenitis is a common occurrence during the progressive phase of the disease.^1^ Inorganic birefringent particles were detected within macrophages in the cortex and medulla of femoral lymph nodes of people living in endemic areas, both affected and non-affected.^5^ Furthermore, Price verified these particles to be predominantly formed of silica, aluminium and iron, presumably a form of silicate.^6^

Only a subset of exposed people develop podoconiosis. In a pedigree study conducted in Ethiopia, Price first proposed the existence of a genetic component to susceptibility to podoconiosis.^7^ A more recent study in Ethiopia estimated the heritability of podoconiosis to be 63%, with a sibling recurrence risk ratio of 5.07.^8^ Age, and a history of consistent use of footwear, were established as environmental covariates.^8^ A genome-wide association study (GWAS) conducted in a population from southern Ethiopia revealed that genetic variants located in the region encompassing the HLA class II (HLAII) genes *HLA-DRB1, HLA-DQA1* and *HLA-DQB1* significantly predispose to the disease.^9^ These findings have now been replicated in a second GWAS in an independent larger data set comprising three Ethiopian ethnic groups (T. Gebresilase, unpublished data). Whether the HLA molecules encoded by these genes have a functional role in podoconiosis is not yet determined, but there are examples of diseases triggered by minerals for which HLAII molecules play a direct role in pathogenesis. For example, HLA-DP alleles carrying a glutamic acid at position 69 are able to present an unknown antigenic form of beryllium to beryllium-specific T cells, and are associated with beryllium sensitisation and the development of chronic beryllium disease.^10^ The HLAII association suggests that T cell inflammatory response(s) may be critical in the development of podoconiosis^9^ and further research is required to understand the molecular pathways involved. In addition to more detailed genetic mapping and HLA typing studies that aim to identify the disease-causing variants, gene expression studies can also be helpful to identify the key molecules involved.

Price's extensive work on podoconiosis in Ethiopia in the 1970s and 1980s resulted in a significant collection of materials, including formalin-fixed and paraffin-embedded (FFPE) patient tissue blocks, slides and notebooks. This archive (rediscovered in 2013 and currently held at Brighton and Sussex Medical School, UK) provides an invaluable source material and a starting point for present-day evaluation of the morphologic and immunophenotypic features of podoconiosis.^11,12^ Histopathological analysis of these FFPE lymph nodes and skin blocks confirmed extensive fibrosis and lymph node follicular hyperplasia with an increased lymphocytic population, which was found to be composed mostly of CD4^+^ T lymphocytes.^11^ Histopathologic changes were also observed in skin nodules of podoconiosis patients with advanced-stage disease.^13^ Extensive sclerosis, verrucous acanthosis (not HPV-induced) and loss of elastic fibres was observed, as well as increased lymphocytic infiltration, particularly by T cells, and also by mast cells and macrophages.^13^

Identifying differentially expressed genes and pathways is a key focus of translational research. Due to various constraints regarding ethics considerations and limited services in the remote areas where affected individuals reside, it is difficult to procure fresh tissue samples, which is why for the present study we turned to the available fixed material in Price's archive. Formalin fixation and paraffin embedding has long been used in pathology labs as a practical and effective method of preserving and storing clinical samples, and historical FFPE archives often comprise unique specimens that are difficult to replicate today due to changes in medical practice. However, downstream molecular analysis of FFPE-derived nucleic acids can be challenging, as RNA from FFPE samples continues to degrade with time.^14^ Often this RNA is not suitable for RNA-sequencing, traditional microarray or quantitative PCR (qPCR).^15^ The NanoString nCounter platform offers advantages for retrospective clinical studies mainly in that RNA content is measured directly using molecular ‘barcodes’ and single molecule imaging. It does not require amplification or other enzymatic processing, allowing gene expression signatures to be identified even when nucleic acids are degraded.^15^ Sensitivity is comparable with qPCR.^16^ Only small tissue samples are required and hundreds of target genes can be processed in a single reaction, generating high-quality transcriptomic data.^15^ Good correlations have been observed comparing samples from FFPE archived material and fresh (frozen) samples, although the number of genes detected from FFPE material is generally lower.^15^ Taking these advantages and limitations into consideration, we determined that NanoString was the most appropriate technique with which to explore the nearly 5-decade-old unique material included in Price's historical podoconiosis archive and perform the first multiplex characterisation of gene expression in podoconiosis from femoral lymph nodes of affected individuals from Ethiopia. Analysis of differentially expressed genes conducted in this proof of concept study could give insights into biological pathways or mechanisms associated with podoconiosis and serve as the basis for further research towards treatments and the development of diagnostic tests for podoconiosis.

## Materials and Methods

### Specimen selection

FFPE tissue blocks were selected that appeared well preserved and clearly labelled as either from a patient with podoconiosis or a normal control. These samples were dated from 1970 to 1976 and were collected in Ethiopia. A range of different patient clinical presentations was selected, labelled by Price as ‘swollen leg’ (SL), ‘water-bag’, ‘rubbery’ and ‘wooden’ types (Table 1). The samples selected included femoral lymph nodes (either the whole node or sections) and skin samples. After discarding the outer layers, very thin slices of the material were cut from the FFPE blocks manually using scalpels and immediately transferred to Eppendorf tubes for the RNA extraction protocol to minimise exposure to air and light.

**Table 1.** Clinical information for 15 subjects included in this study and quality parameters of RNA extracted from archival formalin-fixed and paraffin-embedded (FFPE) lymph node blocks

Clinical information						RNA quality				
FFPE block	Type	Age	Gender	Notes by E. Price	Date	RNA, ng/ul	A260/280	A260/230	RIN	50–300, nt
621B	Podo	n/a ‘young’	M	SL up to knee, whole foot covered with large ‘nodules’; skin very tough to cut. Part of node from left groin with lymph vessels entering. On 2/8/71 leg amputated below knee	1970	61.0	1.8	1.1	2.4	71%
764	Podo	33	M	Right femoral node WB leg; node embedded in fat	1972	63.7	1.8	1.5	2.4	70%
795	Podo	22	F	Bilateral SL (rubbery); right femoral node, contains one white spot of about 0.25 cm (necrosis?)	1973	28.6	1.8	1.9	2.4	62%
821	Podo	18	M	One-sided left SL WB only up to ankle; left inguinal node with femoral ‘tail’	1973	32.6	1.9	1.4	2.4	66%
826	Podo	46	F	WB, right femoral node; node embedded in fat	1973	8.7	1.7	1.4	n/a	71%
828	Podo	17	M	Left femoral node; an early SL (WB). Node small but very fibrous + ‘should, I think, contain granulomata of early disease’	1973	29.4	1.9	1.5	2.4	64%
830	Podo	16	M	Right femoral node; 2 wk after acute attack; the node was still hyperaemic + very adherent + ‘should be interesting’	1973	11.8	1.8	1.2	2.4	76%
833	Podo	n/a	M	Left femoral node. Big SL smooth rubbery. Two large adherent nodes. This patient had much infection of toe clefts of this foot on arrival	1973	54.6	2.0	1.6	1.3	73%
843	Podo	18	F	Right femoral node; wooden type. Both nodes were very fibrous 2×1 cm embedded in fibro fatty tissue	1973	2.8	1.9	1.5	1.1	78%
849	Podo	25	F	Right femoral node; rubbery type both sides. Both sides very adherent to surrounding fatty tissue	1974	2.4	1.9	1.6	1.1	69%
850	Podo	13–15	M	Right femoral node; one-sided right full-length SL (4 y with tuberculae). Multiple scars in right groin? The node was very adherent + uniformly fleshy on section	1974	30.5	2.0	1.8	2.4	74%
895	Control	n/a	n/a	Autopsy normal control node; femoral region	1976	62.0	1.9	1.7	2.5	80%
896B	Control	n/a	n/a	Autopsy normal control node; femoral region	1976	27.4	1.8	1.8	2.4	81%
897	Control	n/a	n/a	Autopsy normal control node; femoral region	1976	70.9	1.8	1.8	2.5	85%
898	Control	n/a	n/a	Autopsy normal control node; femoral region	1976	53.1	1.9	1.6	2.5	78%

Abbreviations: F, female; M, male; Podo, podoconiosis case; RIN, RNA integrity number; SL, swollen leg; WB, ‘waterbag’ type.

### RNA purification

Thinly cut sections of FFPE specimens were de-paraffinised and total RNA was extracted using the RNeasy DSP FFPE Kit (Qiagen, Hilden, Germany) according to the manufacturer's instructions. This kit was chosen as it showed increased performance for extraction of RNA from archival FFPE material.^15,17^ RNA integrity number (RIN) scores were obtained using the Agilent 2100 Bioanalyzer Eukaryote total RNA 6000 Pico chip (GCB, Durham, NC, USA). RNA quantity and purity were assessed using a NanoDrop spectrophotometer (ThermoFisher Scientific, Waltham, MA, USA).

### NanoString nCounter assay

All samples were analysed using the nCounter Human Immunology V2 panel (comprising 579 general immunology-related genes) on a NanoString nCounter FLEX platform (NanoString, Seattle, WA, USA) in accordance with the manufacturer's guidelines. The platform operates by a solution-based mRNA hybridisation of short length probes, which carry a streptavidin component, that become fixed to a cartridge coated in biotin. Digital imaging of the cartridge is then used to quantify expression. The samples used in this study were sent to Newcastle NanoString Facility (Newcastle University, Newcastle, UK) for processing. In brief, 5 µL of sample (25–50 ng/µl) was combined with hybridisation buffer and probes (reporter and capture) before hybridisation at 65°C for 24 h. All samples were then processed with the NanoString Prep station. Cartridges were scanned on the Nanostring Digital Analyzer to generate a data set of reporter code counts (RCC), serving as a relative quantification of gene expression.

### Data normalisation and statistics

The RCC were collected using nSolver Analysis Software v.4.0 (NanoString, Seattle, WA, USA). A quality check of raw data was performed using the same software. A heatmap of all data was obtained using nSolver Advanced Analysis. This heatmap was generated via unsupervised clustering using the geNorm algorithm for housekeeping (HK) gene selection, scaled to give all genes equal variance. Due to the aged condition of the RNA input, a filtering strategy was adopted for differential gene expression analysis to remove low signal data that could be due to degradation. A background threshold was defined for each sample based on the mean +2 SD of eight negative controls. HK genes whose raw probe counts were close to background were removed from the normalisation process. A panel of four HK genes showed robust signal and good expression correlation (EEF1G, GAPDH, POLR2A, RPL19) and were manually selected to normalise this data set in nSolver. Low count genes were then filtered out based on a defined signal-to-noise ratio: signal divided by background, using each sample's own background signal. Targets were scored as present that had a signal-to-noise ratio >3. Unpaired *t*-tests were performed to compare expression between groups (podoconiosis vs normal controls); p-values presented were not corrected for multiple comparisons as our priority was to minimise type 2 error. Differentially expressed genes (DEGs) were defined as those with a fold change of ±1.5 and uncorrected p<0.05. GraphPad Prism8 (GraphPad Software, San Diego, California, USA) was used for statistical analysis.

### Gene ontology and pathway enrichment analysis

Biological functions of differentially expressed genes between podoconiosis and control samples were explored using online tools in Metascape (http://metascape.org/gp/index.html#/main/step1).^18^ Functional enrichment in the molecular function category was performed using gene ontology (GO) terms. Pathway enrichment analysis was performed using terms from the following databases: Kyoto Encyclopedia of Genes and Genomes, GO terms in biological processes, Reactome Gene Sets, Canonical Pathways and Comprehensive resource of mammalian protein complexes. Input data comprised the list of upregulated and downregulated genes identified in the differential expression analysis. Protein-protein interaction networks were evaluated using the Search Tool for the Retrieval of Interacting Genes Database (STRING v11.0, https://string-db.org/).^19^

## Results

### RNA quality

Purified RNA samples were successfully obtained from femoral lymph node blocks of podoconiosis patients and controls. None of the skin specimens yielded sufficient RNA and therefore were not included. Of the 32 samples analysed transcriptomically, 15 passed NanoString QC parameters after sample processing. RNA quality parameters for the 15 samples are summarised in Table 1. As expected, the RNA obtained from the aged material was highly degraded. RIN scores varied from 1.1 to 2.5 (a RIN score of 10 meaning highest integrity and 1 referring to highly degraded RNA) and electropherograms did not show clearly visible ribosomal RNA peaks (not shown). Low RIN scores are considered typical for archival FFPE extractions.^20^ Furthermore, 62–85% of the RNA samples contained fragments measuring between 50–300 nucleotides in length. This is also consistent with archival material.^21,22^ RIN scores obtained by the BioAnalyzer did not correlate with successful nCounter results in this case, as some RNAs with higher RIN scores showed a low binding affinity in the nCounter assay. The low A260/230 ratios obtained may be indicative of the presence of organic contaminants in the extracts resulting from fixation/RNA extraction protocols.

Table 1 also summarises patient classification and descriptions of clinical features as extracted from Price's notebooks that accompanied the well-catalogued collection. Of the two main clinical types, ‘water-bag’ refers to a presentation that is mainly lymphoedematous with limited fibrotic thickening and hyperkeratosis, whereas the ‘leathery type’ refers to hard skin due to fibrosis and prevalence of hyperkeratosis. This descriptive classification reflects the wide range of presentations that accompany podoconiosis disease progression to elephantiasis, yet it does not conform to the current clinical staging system used for podoconiosis as this was not developed until 2008. The modern system encompasses five stages based on disease severity with 1 being the least severe and 5 being the most advanced stage.^23^ In most cases it was not possible to correlate the descriptions found in Price's notes to the current staging system. The SL descriptor used by Price, for example, is non-specific and can be observed at different stages and therefore is not a good indicator of disease severity.

### Gene expression analysis

**Figure 1.**
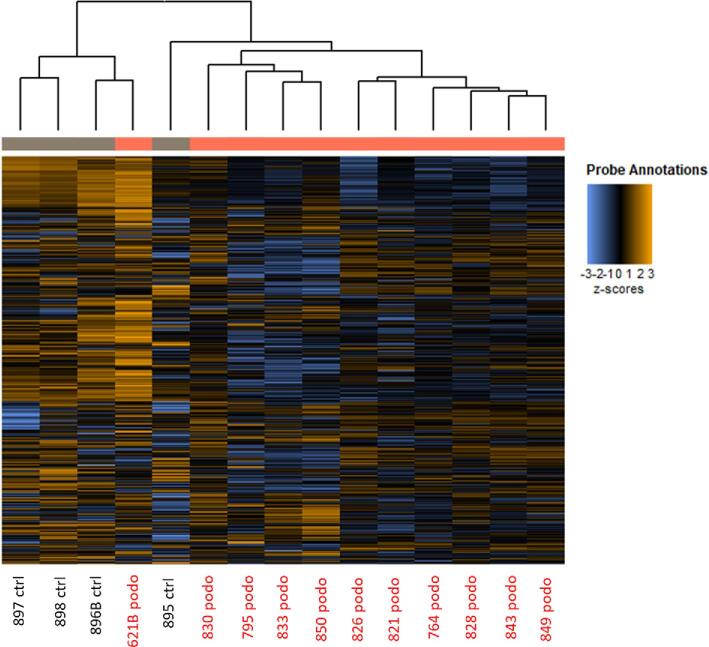
Heatmap clustering of gene expression in archival lymph nodes samples for podoconiosis patients and controls. Unsupervised hierarchical clustering based on gene expression data for 11 podoconiosis samples and 4 control samples. Rows represent individual genes (log2 counts of 579 gene codeset in nCounter Human Immunology V2 panel). Orange indicates high expression and blue indicates low expression. Heatmap generated using NSolver Advanced Analysis software.

NanoString gene expression profiling was performed on RNA extracted from 15 archival FFPE samples using the complete nCounter Human Immunology V2 codeset. A high level exploratory view of the data showing relative expression levels of 579 immunology genes is shown in Figure 1. FFPE-derived RNA samples obtained from podoconiosis patients cluster together as expected, with the exception of sample 621B, which is classified as nodular SL but clustered with the normal control samples. Nevertheless, and despite the broad range of disease severities and heterogeneity of clinical pathological traits, this cluster analysis revealed a clear separation between samples from affected and unaffected individuals.

**Figure 2.**
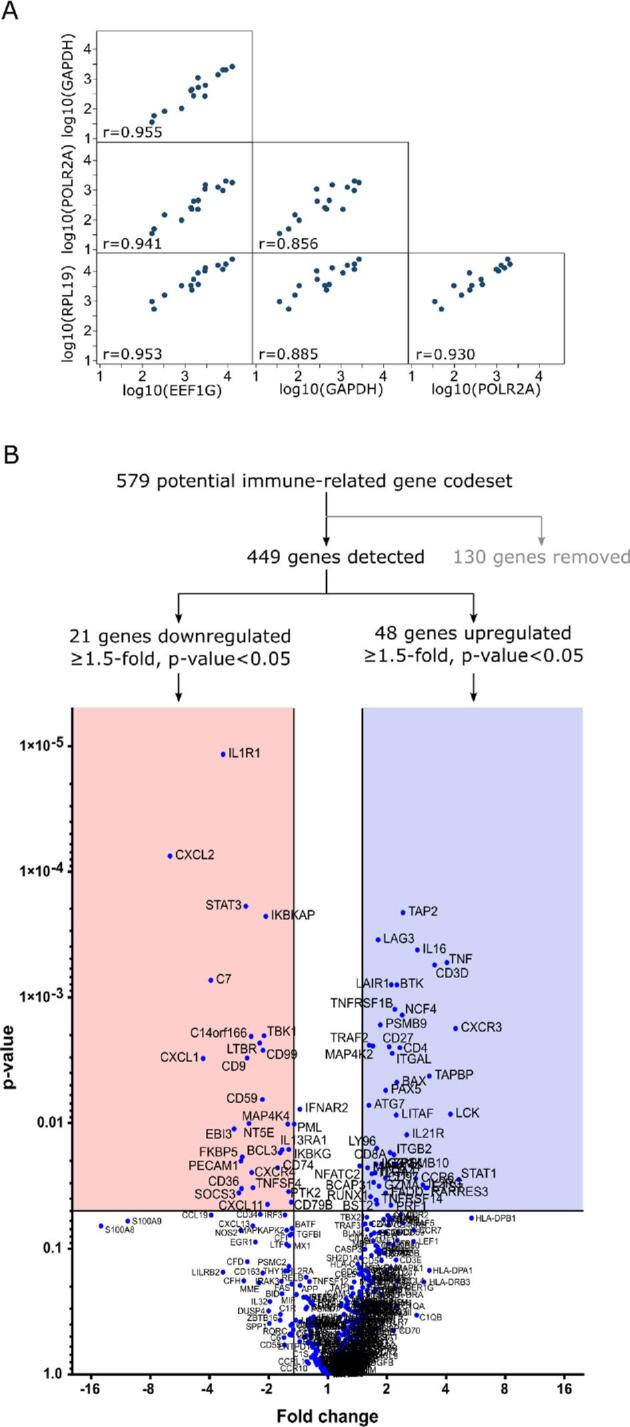
Analysis of the differential expression of genes in archival FFPE lymph nodes of podoconiosis patients and controls. (A) Scatter plot matrix and Pearson correlation coefficients for the selected housekeeping genes used to normalise the data for DE exploration. (B) Diagram depicting gene selection outcomes and volcano plot analysis of DE genes between podoconiosis and control samples. The log2-fold change values are plotted on the x-axis of the volcano plots and are compared with the negative log10 p-values on the y-axis.

**Figure 3.**
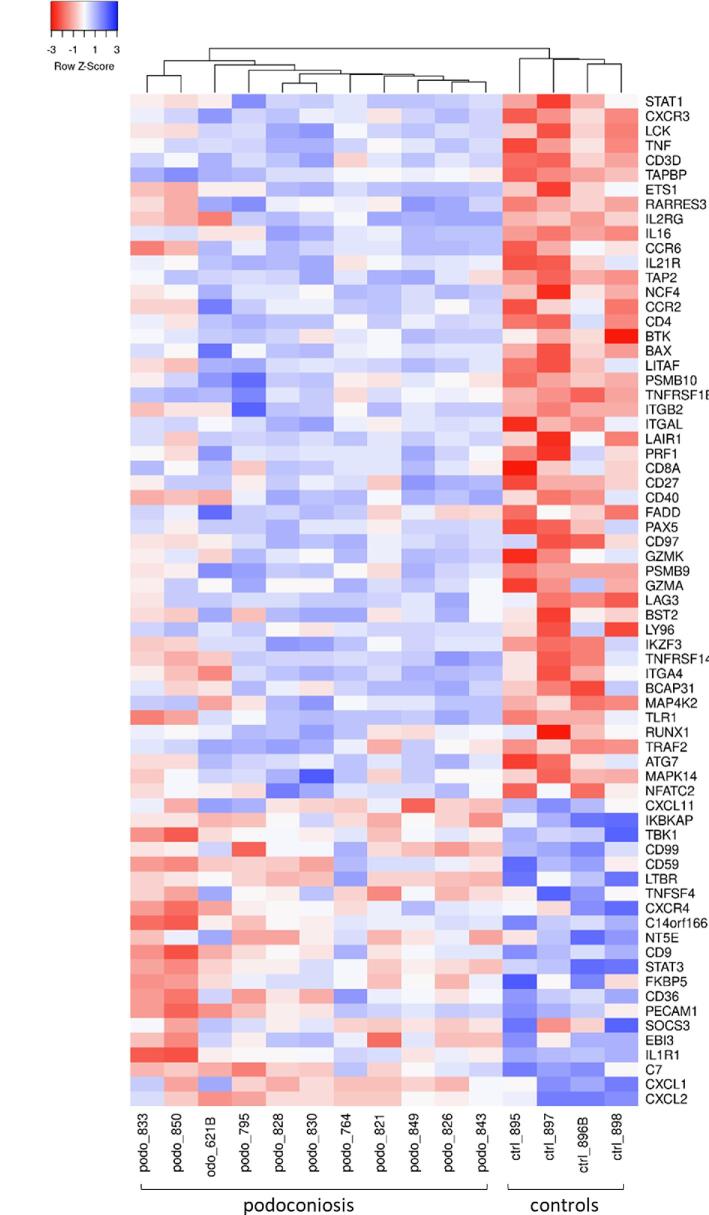
Heatmap of differentially expressed genes between podoconiosis cases and normal controls. Hierarchical clustering based on Euclidean distance. Normalised and log2-transformed data were used to create the heatmap. The genes are listed in the rows and the podoconiosis and control groups are listed on the bottom. Colours indicate scale of gene expression (blue: upregulation; red: downregulation).

For differential gene expression evaluation, a careful analysis of the data was performed to filter out low quality data due to sample degradation, because of the difficulty in distinguishing genes expressed at low levels from degraded RNA. Four HK genes with high expression levels and good correlation (Pearson correlation coefficients between 0.856 and 0.955) were selected to normalise the data for DEG analysis (Figure 2A). Genes expressed at very low levels and genes with low RCC counts (signal-to-background ratio <3) were excluded from the analysis. The resulting NanoString gene expression profiles of 11 podoconiosis cases were then compared with 4 normal controls. Of 449 detected immune-related genes, 48 genes were upregulated and 21 genes downregulated (p<0.05; expression fold change [FC]≥1.5) in the podoconiosis group compared with the control group (Figure 2B; Table 2). An overview of gene expression changes between podoconiosis cases and normal controls is presented in a heatmap format (Figure 3).

**Table 2.** List of differentially expressed genes in podoconiosis archival formalin-fixed and paraffin-embedded (FFPE) lymph nodes. DE gene data based on 11 patients and 4 normal controls using the nCounter Human Immunology V2 panel, with p<0.05 and FC≥1.5

Gene	FC	p-value	Gene	FC	p-value
Upregulated					
*STAT1*	4.6	0.028	LY96	1.8	0.016
*CXCR3*	4.5	0.002	IKZF3	1.8	0.021
*LCK*	4.2	0.008	TNFRSF14	1.8	0.041
*TNF*	4.0	0.001	ITGA4	1.7	0.025
*CD3D*	3.5	0.001	BCAP31	1.7	0.030
*TAPBP*	3.3	0.004	MAP4K2	1.7	0.002
*ETS1*	3.2	0.033	TLR1	1.7	0.025
*RARRES3*	3.1	0.033	RUNX1	1.6	0.038
*IL2RG*	3.0	0.031	TRAF2	1.6	0.002
*IL16*	2.9	<0.001	ATG7	1.6	0.007
*CCR6*	2.8	0.028	MAPK14	1.6	0.023
*IL21R*	2.5	0.012	NFATC2	1.5	0.022
*TAP2*	2.4	<0.001			
*NCF4*	2.4	0.001			
*CCR2*	2.3	0.054	Downregulated		
*CD4*	2.3	0.003	CXCL2	−6.4	<0.001
*BTK*	2.2	0.001	CXCL1	−4.3	0.003
*BAX*	2.2	0.005	C7	−3.9	0.001
*LITAF*	2.2	0.009	IL1R1	−3.4	<0.001
*PSMB10*	2.2	0.021	EBI3	−3.0	0.011
*TNFRSF1B*	2.2	0.001	SOCS3	−2.8	0.036
*ITGB2*	2.2	0.018	PECAM1	−2.8	0.020
*ITGAL*	2.1	0.003	CD36	−2.7	0.033
*LAIR1*	2.1	0.001	FKBP5	−2.7	0.019
*PRF1*	2.1	0.045	STAT3	−2.6	<0.001
*CD8A*	2.1	0.017	CD9	−2.6	0.003
*CD27*	2.1	0.002	NT5E	−2.5	0.010
*CD40*	2.0	0.054	C14orf166	−2.5	0.002
*FADD*	2.0	0.036	CXCR4	−2.4	0.025
*PAX5*	2.0	0.005	TNFSF4	−2.4	0.033
*CD97*	2.0	0.027	LTBR	−2.2	0.002
*GZMK*	1.9	0.021	CD59	−2.2	0.006
*PSMB9*	1.8	0.002	CD99	−2.1	0.003
*GZMA*	1.8	0.032	TBK1	−2.1	0.002
*LAG3*	1.8	<0.001	IKBKAP	−2.1	<0.001
*BST2*	1.8	0.044	CXCL11	−2.0	0.044

Abbreviation: FC, expression fold change.

### Pathway analysis

**Figure 4.**
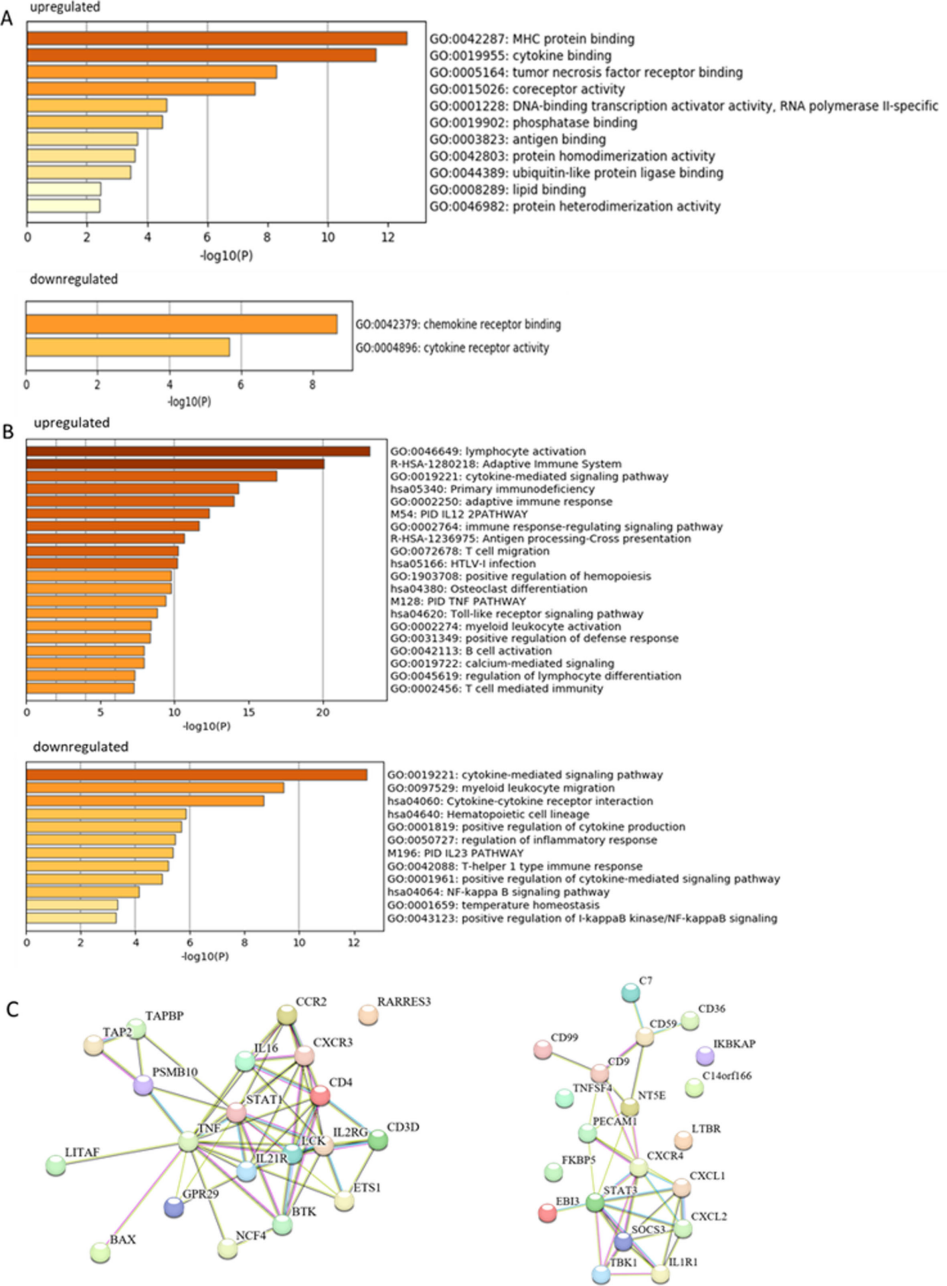
Functional enrichment analysis of differentially expressed genes in podoconiosis FFPE lymph nodes compared with controls. (A) Heatmap of enriched terms in gene ontology (GO) molecular function category among upregulated and downregulated genes in podoconiosis. (B) Heatmap of pathway and process terms enrichment analysis of upregulated and downregulated genes in podoconiosis. The x-axis indicates significance level (scored as –log10 (p-value); (C) interaction network of top 20 upregulated (left) and downregulated (right) genes in podoconiosis, displaying linked-up genes that participate within the same biological pathway.

GO and pathway enrichment analyses were performed based on the list of DEGs to clarify the biological functions and pathways that contribute to the difference in expression patterns observed between lymph node samples of podoconiosis patients and controls. Molecules involved in MHC, cytokine and TNF receptor binding, coreceptor activity and transcription activation were the most significantly upregulated in podoconiosis (Figure 4A). By contrast, the 21 downregulated DEGs in podoconiosis are involved in chemokine and cytokine receptor activity. Pathway analysis showed significantly upregulated genes in podoconiosis to be related to lymphocyte activation, the adaptive immune response, cytokine signalling, IL-12 pathway and antigen processing (Figure 4B). The list of significantly downregulated genes was most related to cytokine signalling, myeloid leukocyte migration, the regulation of inflammatory response and IL-23 pathway. The top 20 differentially expressed proteins, in particular the top ones overexpressed, are highly interactive, as depicted in the overview of the known physical and functional connections between the proteins (Figure 4C).

## Discussion

Podoconiosis is a debilitating condition that affects some of the most vulnerable populations in tropical regions of Africa, Central and South America and Southeast Asia.^24^ Although it has been well characterised as a separate entity from lymphatic filariasis (LF) for several decades, it has only recently started to receive significant attention. Understanding the immunopathogenesis of podoconiosis is crucial for the improvement of treatments that could benefit the millions of people currently affected, as well as for the development of a diagnostic test that could differentiate it from other forms of tropical lymphoedema, including infectious diseases such as LF and leprosy that require different treatment regimens.

Pioneer work into the histopathological characterisation of this condition was performed in the 1970s and 1980s by Ernest Price^5,6^ and, at present, FFPE tissues belonging to Price's historical collection are the only podoconiosis tissue samples available to us. Attempting to unlock gene expression data from decades-old FFPE is necessarily accompanied by concerns regarding RNA quality and integrity. Formalin fixation is well known to result in modifications such as RNA fragmentation and cross-linking to proteins. Additionally, other parameters can further affect the quality of extracted RNA such as the time between sample retrieval and fixation, the fixation and paraffin embedding procedures themselves, as well as storage time and storage conditions of the FFPE blocks. The fact that the control nodes were labelled by Price as autopsies, whereas the patient-derived tissues were assumed to be biopsy material may imply that there were delays in fixation of the controls. Apart from the long storage time, we have no information regarding these parameters, so suitability for downstream molecular applications can only be assessed after extracting the RNA and while performing the assays. However, it is worth mentioning that even though parameters such as RNA yield and fragmentation indicators (such as RIN and fragment size) were low, they were comparable between the cases and control samples, suggesting that differences in fixation may not necessarily have been a factor in the outcomes obtained for these samples. In view of these limitations, we chose the NanoString platform for differential gene expression analysis because it relies on direct hybridisation of small probes and does not require amplification of the genetic material, which is an advantage when relying on low quality and/or low input RNA samples. As such, it is often the method of choice to generate high-quality, high-throughput expression data from clinical archival FFPE material.

We extracted mRNA from 5-decade-old FFPE tissue samples for analysis using this technology. Our results using the nCounter Immunology panel indicated that the immune signature detected in podoconiosis patient lymph nodes is distinct from that of the controls, as demonstrated by hierarchical clustering. The sample is not large enough to allow stratification of patient samples according to disease presentation, age or gender. Furthermore, another limitation here is the fact that the disease descriptors used by Price do not match the current podoconiosis clinical staging system and therefore sample sorting according to disease presentation/staging was not possible. As such, we elected to group and analyse the samples merely based on the presence/absence of podoconiosis.

Among the differentially upregulated genes, significant biological functions identified relate to cytokine, MHC and TNF receptor binding, coreceptor activity and transcription activation. Furthermore, integrated pathway analysis revealed an association with lymphocyte activation, the adaptive immune response, cytokine signalling, the IL-12 pathway and antigen processing and presentation.

Unfortunately, expression of the various HLAII genes found to be genetically associated with podoconiosis was not detected in this analysis, as that group of genes was removed from the batch after our strict ‘low count’ quality control, which cannot be distinguished from degraded target.

Nevertheless, a significant increase in expression of genes related to MHC binding and antigen processing still supports the hypothesis that antigen-driven T cell responses may play a key role in the pathogenesis of podoconiosis. MHC molecules can play a direct role in driving the immune response against metal ions. In chronic beryllium disease, one of the best studied metal-induced hypersensitivity conditions, specific MHCII/peptide/metal coordinated structures are presented by activated dendritic cells and can be recognised by T cell receptors.^10^ T cell activation with accompanying secretion of IL-2 results in clonal expansion of beryllium-reactive CD4^+^ T cells, which can be readily reactivated upon reexposure to the metal, causing further cell expansion, release of cytokines and chemokines, cell damage and triggering inflammation.^25^ A similar scenario could be at play in the case of podoconiosis in the response to a soil mineral/inorganic component.

Additionally, CD4^+^ T cells have been implicated in the development of secondary lymphoedema. An increased number of tissue-infiltrating CD4^+^ T cells were observed both in lympheodema mouse models as well as in cancer-related lymphedema, and CD4^+^ T cell depletion in mice prevented the development of lymphoedema (but not CD8^+^ T cell or macrophage depletion).^26^ Naive CD4^+^ T cells were shown to be activated in skin-draining lymph nodes following interaction with antigen-presenting cells, after which they migrate to the skin.^27^ Expression of vascular endothelial growth factor C was induced by activated CD4^+^ T cells resulting in dysregulated lymphangiogenesis, whereby the formation of immature and leaky lymphatic vessels led to the accumulation of interstitial fluid. Additionally, decreased levels of extracellular matrix markers such as E-cadherin, type III collagen and TGF-β1 were observed after CD4 depletion in the lymphatic stasis mouse model, associated with reduced fibrosis.^28^ Although lymphoedema is characterised by a mixed Th1/Th2 type infiltrate, a biased Th2 response has been observed in mouse lymphoedema models and breast cancer-related lymphoedema.^26^ CD4^+^, IL-4^+^, IL-13^+^ and Th2 cells are believed to play a crucial role in the regulation of fibrosis, with IL-4 and IL-13 having been shown to impair lymphatic endothelial cell survival and proliferation.^29^

Due to the retrospective nature of this study, another limitation was the inability to assess the performance of this assay on archival FFPE material in comparison with fresh tissue. Alhough fresh frozen tissue was unavailable to us, good correlations have been observed in numerous other studies involving archival FFPE, particularly when using NanoString technology, which is encouraging.^15,20^

Although the data presented here are descriptive and require validation in larger cohorts and fresh tissue, they demonstrate the potential to use precious archival material to identify gene signatures and provide information on immunological changes related to podoconiosis. Furthermore, these findings may serve as baseline data for further research into the poorly understood immunopathogenesis of this disease. For instance, investigating whether these lymph node findings correlate with expression in peripheral blood would be an important step towards developing a point of care test for the diagnosis of podoconiosis.

### Conclusion

This study reports a multiplex gene expression analysis in podoconiosis and provides evidence of upregulation of pro-inflammatory transcripts suggestive of chronic immune activation in this disease. The molecular pathways identified are implicated in podoconiosis and can inform further studies, paving the way for future research into diagnostic and treatment options. In addition, this study also demonstrates the feasibility of employing NanoString technology to detect differentially expressed transcripts in aged archival FFPE lymph node tissue.

## Data Availability

Data are available on request.
